# Electroacupuncture Pretreatment Reduces Ischemic Brain Injury by Inhibiting the Lactate Production and Its Derived Protein Lactylation Formation

**DOI:** 10.1111/cns.70231

**Published:** 2025-01-20

**Authors:** Xin‐Ru Pan, Yao‐Dan Zhang, Yuan‐Hui Gan, Jia‐Hang Zhang, Su‐Jin Gao, Xiao‐Shuang Feng, Jia‐Xin Xie, Yu‐Fei Wang, Xin‐Xiao Zhang, Peng‐Fei Wang, Shu‐Guang Yu, Yong Tang, Xiao‐Yi Xiong

**Affiliations:** ^1^ Acupuncture and Tuina School Chengdu University of Traditional Chinese Medicine Chengdu China; ^2^ Department of Neurology, Weihai Municipal Hospital, Cheeloo College of Medicine Shandong University Weihai China; ^3^ Sichuan Provincial Key Laboratory for Acupuncture & Chronobiology Chengdu China; ^4^ Ministry of Education Key Laboratory of Acupuncture for Senile Disease (Chengdu University of TCM) Chengdu China; ^5^ International Collaborative Centre on Big Science Plan for Purinergic Signalling Chengdu University of Traditional Chinese Medicine; School of Health and Rehabilitation, Chengdu University of Traditional Chinese Medicine Chengdu China

**Keywords:** electroacupuncture pretreatment, glycolysis, ischemic stroke, lactate, lysine lactylation

## Abstract

**Aim:**

Given that electroacupuncture (EA) pretreatment inhibits lactate production and lactate‐derived lysine lactation (Kla) aggravates ischemic brain injury, we aimed to investigate whether the formation of Kla protein is involved in EA pretreatment to alleviate ischemic brain injury.

**Methods:**

EA was performed on the Baihui acupoint (GV20) of male C57BL/6J mice before receiving the permanent middle cerebral artery occlusion (pMCAO) surgery. Western blot and immunofluorescent staining were used to observe neuronal survival, astrocyte activation, and protein Kla levels, and the lactate levels in ischemic brains were assayed with a commercial kit. TTC staining and neurological function scores are performed to evaluate the brain damage in mice.

**Results:**

We found that the increased lactate content and protein Kla levels were significantly decreased in ischemic brain tissue of mice after receiving EA pretreatment, and accompanied by the reduction of astrocyte activation and neuronal injury and death. Meantime, we found that EA pretreatment was effective in reversing the worsening of ischemic brain injury caused by lactate supplementation. However, EA pretreatment did not further reduce the lactate content and protein Kla levels and ameliorate brain injury in ischemic stroke mice after inhibition of glycolysis.

**Conclusion:**

Our study reveals that EA pretreatment reduced ischemic brain damage by inhibiting lactate production and its derived protein Kla formation in mice with ischemic stroke.

## Graphical abstract

Electroacupuncture pretreatment can inhibit the glycolysis process in astrocytes after ischemia, reduce the activation of astrocytes, and reduce the lactate‐derived lysine lactation level, thereby reducing neuronal damage and improving ischemia‐induced brain injury.

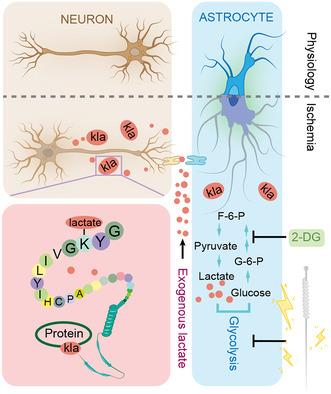

## 1 Introduction

In recent years, the lifetime risk of stroke has increased due to severe aging and the accumulation of risk factors, and 87% of all strokes are ischemic [1]. Although endovascular therapy has been used as a current main therapy for its remarkable efficacy [2, 3], the narrow time window [4] excludes most patients of acute ischemic stroke (~93%) from this approach [5]. However, there are still no effective neuroprotective ways that have been used in patients with stroke. Then, it is necessary to further explore the mechanism of brain injury of cerebral infarction and find novel potential therapeutic targets to enhance the ischemic tolerance of brain tissue to extend the narrow time window of endovascular therapy for more patients via reducing the degree of ischemic brain injury. Therefore, if there is a way to increase the tolerance of ischemic and hypoxic injury in brain tissue before the onset of cerebral infarction, it will help to reduce the degree of brain injury.

Acupuncture, a traditional Chinese therapeutic method, has been widely used for the treatment of ischemic stroke and its complications. Multiple clinical and animal studies have shown that acupuncture can alleviate ischemic brain injury [6, 7, 8] via multiple mechanisms, such as reducing inflammation [9, 10, 11], inhibiting cell apoptosis [12, 13], enhancing neuronal synaptic plasticity [14, 15], improving immune function [16], etc. In ancient Chinese medical literature and numerous modern studies, Baihui acupoint (GV20) is one of the most commonly used acupoints for treating ischemic stroke. GV20 is the 20th acupuncture point on the Du Mai, which is the intersection of multiple “yang” meridians, and stimulating it can treat a variety of neurological diseases. A systematic meta‐analysis study showed that the head acupuncture treatment based on GV20 had a significant effect on improving infarct volume [17]. Multiple free randomized controlled trials have found that stimulating GV20 can improve cognitive dysfunction, etc. [18, 19]. In addition, many studies have shown that stimulating GV20 can improve neurological function through anti‐inflammatory effects, regulation of cerebral vascular blood flow, and other mechanisms [20, 21, 22]. In addition, our previous study has shown that electroacupuncture (EA) pretreatment on GV20 also protects against ischemic stroke by regulating brain energy metabolism, like inhibiting glycolysis in ischemic brains [23]. However, the downstream mechanisms by which EA pretreatment reduces brain damage after inhibiting lactate production are still unclear.

The brains of humans and other mammals are highly vulnerable to interruptions in blood flow and decreases in oxygen levels [24], therefore, the disruption of brain energy metabolism is one of the main initial pathological changes in ischemic stroke [25]. Because brain tissues consume large amounts of energy during normal functions, while little energy substrates are stored in brain tissues with limited glycogen reservoir in astrocytes [26]. Then, once cerebral ischemia occurs, the ischemic brain tissue suffers the same damage as “drought” resulting in neuronal dysfunction. Interestingly, one study of global ischemia of pig brains showed that intact large mammalian brain possesses an underappreciated capacity for restoration of microcirculation and molecular and cellular activity after a prolonged postmortem interval [24], suggesting that there may exist some ways or targets to improve the brain tolerance from long‐time ischemia exposure.

After ischemia, neural cells are under a hypoxic environment, which leads to normal energy metabolism being disrupted. For example, the energy metabolism of cells based on oxidative phosphorylation will transfer to that of glycolysis after cerebral ischemia. Because significantly increased lactate levels in ischemic brain tissues have been observed both in patients [27, 28, 29] and animals [30, 31, 32] with ischemic stroke. In recent years, increasing evidence suggests that lactate has multiple roles in involving biological processes [26]. Among these roles, driving the formation of protein lysine lactylation (Kla) is a newly identified protein posttranslational modification (PTMs) [33]. In our recent research works, we found that the increased lactate and protein Kla levels could exacerbate ischemic brain injury [26]. Considering that EA preconditioning could inhibit glycolysis of ischemic brain tissues to offer neuroprotection, whether the protein Kla formation is the mechanism involved in reducing ischemic brain injury pretreated by EA remains unknown.

Therefore, in this study, we aimed to investigate whether the protein Kla formation is also involved in the reduction of ischemic brain injury pretreated by EA. The results showed that EA pretreatment markedly decreased the brain lactate levels and protein Kla levels, accompanied by the reduction of neuronal injury and death, astrocyte activation, and infarcted volume in mice with pMCAO models. Furthermore, EA pretreatment can also reduce the lactate supplementation‐induced aggravation of ischemic brain injury, while it failed to further reduce the ischemic brain injury of stroke mice after 2DG pretreatment. Our study reveals a novel therapeutic mechanism that EA pretreatment offers neuroprotection for mice with ischemic stroke via inhibiting the formation of protein Kla.

## 2 Materials and Methods

### 2.1 Animals and Groups

C57BL/6 male mice (8 weeks, 23 ± 2 g) used in experiments were purchased from Gempharmatech Co. Ltd. (Chengdu, China). The mice were housed in an environment with a 12‐h light–dark cycle and suitable temperature (25°C) and humidity (> 30%), and received food. The mice were randomly divided into different groups after acclimatization time. This study was approved by the Institutional Animal Care and Use Committee of the Institute of Model Animals of Chengdu University of TCM. The study followed the ARRIVE guidelines and was performed in accordance with the National Institutes of Health guide for the care and use of Laboratory animals.

### 2.2 EA Pretreatment

EA pretreatment is the same as our previous studies [23]. The Baihui acupoint (GV 20) is located at the intersection of the sagittal midline and the line linking two ears. The whole process of acupuncture was anesthetized with isoflurane. Acupuncture needles (0.16 × 13 mm; Beijing Zhongyan Taihe Medical Instrument Co. Ltd., Beijing, China) were inserted into acupoints at a depth of 2–3 mm. The mice were pretreated with EA at GV 20 for 30 min with a dense wave of 2/15 Hz and the current is 1.5 mA once a day for five consecutive days. According to previously reported methods [34, 35], the sham EA pretreatment is only to insert the acupuncture needles into the skin of the acupoint without other stimulation such as lifting, inserting, twisting and turning, and connecting the current.

### 2.3 The Permanent Middle Cerebral Artery Occlusion (pMCAO) Model

The permanent middle cerebral artery occlusion (pMCAO) model was used to induce a mouse ischemic stroke model [36]. Briefly, 2 h after the end of the last EA treatment, mice are anesthetized with 1% pentobarbital sodium. The middle cerebral artery (MCA) under the translucent skull can be found by making an incision in the line connecting the right ear and the right outer corner of the eye and peeling off the deep temporal muscle. A hole is drilled and the MCA is coagulated at high temperature using an electrocoagulation pen. The procedure was the same in the sham group, but there was no electrocoagulation.

### 2.4 2,3,5‐Triphenyltetrazolium Chloride (TTC) Staining

Take fresh brain tissue and immediately place it in a −20°C freezer for 20 min. Frozen brain tissue was cut into seven coronal brain sections about 1 mm thick, placed in 2% 2,3,5‐triphenyltetrazolium chloride (TTC; Sigma Aldrich, St. Louis, MO, USA) staining solution, and treated at 37°C for 20 min. Infarcted brain tissue is not stained (white) and normal brain tissue is red. The stained brain tissue is placed in paraformaldehyde overnight. After taking pictures, the area of cerebral infarction was calculated using ImageJ software. The infarction volume (%) = the infarction area/the total slices area × 100%.

### 2.5 Lactate Measurement

Lactate Assay Kit (MAK064; Sigma Aldrich, St. Louis, MO, USA) was used to determine lactate content. Lactate assay buffer is added to fresh brain tissue, which is then homogenized and ground, and the supernatant is collected. Transfer the supernatant to a filtered centrifuge tube with a molecular weight cut‐off of 10 kDa and centrifuge at high speed. Fifty microliters of the sample and 50 μL of the Master Reaction Mix were reacted at room temperature for 30 min in the dark, the absorbance value at 570 nm was measured using a microplate reader. The concentration of lactate in the sample to be measured is calculated based on the relationship between the concentration gradient of the lactate standard and the corresponding absorbance value.

### 2.6 Western Blot Analysis

RIPA lysis buffer containing a mixture of protease and phosphatase inhibitors was added to fresh brain tissue and triturated and homogenized, and the supernatant was taken after centrifugation. Protein concentrations were determined with the BCA Protein Assay Kit (23225, Thermo Scientific, USA). An equal amount of protein (20 μg) was extracted from each sample, separated with a 10% Tris‐Gly PAGE gel, and transferred to a polyvinylidene fluoride membrane. After blocking for 2 h in TBST solution containing 5% skimmed milk powder at room temperature, incubate overnight (4°C) with specific primary antibodies: mouse anti‐NeuN antibody (1:1000, ab104224, Abcam, USA), goat anti‐GFAP antibody (1:1000, ab53554, Abcam, USA). Then, wash the membrane five times for 5 min each in TBST. Incubate the secondary antibody corresponding to the primary antibody at room temperature: horseradish peroxidase (HRP) conjugated goat anti‐mouse secondary antibody (1:3000, SA00001‐1, Proteintech, China) or HRP conjugated rabbit anti‐goat secondary antibody (1:3000, SA00001‐4, Proteintech, China). Immunoreactive proteins were visualized using an enhanced chemiluminescent (ECL) substrate (180–501, Tanon, China) and an imaging system (GelView 6000Plus, Guangzhou Biolighting Biotechnology Co. Ltd., Guangzhou, China) after five TBST washes each. After stripping off the membrane, incubate with rabbit anti‐GAPDH antibody (1:1000, 10494‐1‐AP, Proteintech, China). The band intensity was quantified with ImageJ software and normalized to the load control. All Western blot data supporting the results of this study can be checked on in the supplementary information (DataS1).

### 2.7 Immunofluorescent Staining

Take mouse brain tissue fixed by cardiac perfusion, fix it with 4% paraformaldehyde (PFA) for 24 h, and dehydrate it in gradient sucrose. Then, cut the brain tissue into coronal sections with a thickness of 10 μm. The brain slices were washed three times with PBS, each time for 5 min. They were then incubated with blocking perforation solution (0.5% Triton‐X‐100, 5% BSA solution, PBS as the solution) at 37°C for 1.5 h, and then incubated overnight with specific primary antibody: mouse anti‐NeuN antibody (1:200, ab104224, Abcam, USA) and rabbit anti‐ALDH1L1 antibody (1:100, ab87117, Abcam, USA). Then wash away the primary antibody and incubate at room temperature for 2 h for the secondary antibody: FITC‐conjugated goat anti‐mouse IgG antibody (1:200, SA00003‐1, Proteintech, China) or a Cy3‐conjugated affinipure goat anti‐rabbit IgG antibody (1:200, SA00009‐2, Proteintech, China). Capture images using a confocal microscope (Leica) and quantify the intensity of immunofluorescence signals using ImageJ software.

### 2.8 Neurological Function Assessment

To evaluate the neurological function injury and recovery after cerebral infarction in mice, Clark focal functional injury score [37] and glue removal test [38] were used in this study. Mice in the different groups were scored from 9 a.m. to 10 a.m. on the 1st, 3rd, 5th, and 7th day after modeling. The higher the score was, the more serious the neurological function injury was. The total score of all items was recorded for subsequent statistical analysis.

### 2.9 Statistical Analysis

All data were expressed as “mean ± standard deviation (SD)”. All experimental data were statistically analyzed and plotted by GraphPad Prism 8.0 and SPSS25.0 software on a computer. First, we used the Shapiro–Wilk test to detect the normal distribution of the data and then used Levene's test to test the homogeneity of variance for the data conforming to the normal distribution, and the Student's *t*‐test for the data conforming to the normal distribution and homogeneous variance, theWelch's *t*‐test is used for data that conform to the normal distribution but have irregular variance, while the nonparametric test, such as the Mann–Whitney U test or the Kruskal–Wallis *H* test is used for data that do not conform to the normal distribution. *p* < 0.05 was used as the standard of statistical significance.

## 3 Results

### 3.1 EA Pretreatment Mitigated Brain Injury in Mice With Ischemic Stroke

**FIGURE 1:**
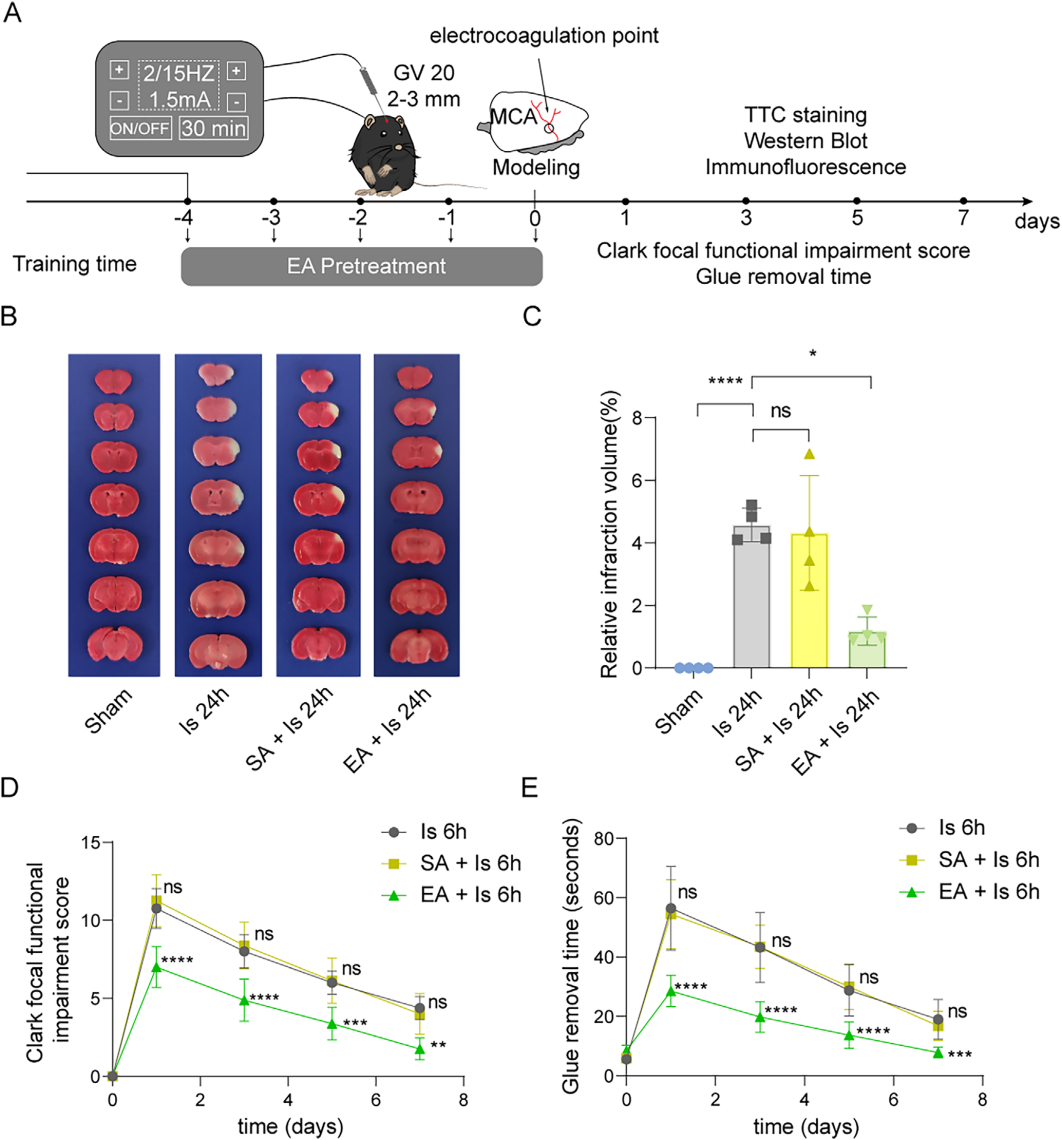
EA pretreatment mitigated brain injury in mice with ischemic stroke. (A) The flowchart shows the modeling method, the timeline of EA pretreatment, and the entire experimental design. (B) Representative images of 2,3,5‐triphenyltetrazolium chloride (TTC) staining of sham, ischemic 24 h, SA (sham EA) pretreatment + ischemic 24 h, and EA pretreatment + ischemic 24 h. The infarction area is white (*n* = 4). (C) Statistical chart of the proportion of cerebral infarction area. (D, E) Mice with EA pretreatment and ischemic stroke show neurological function in tests of (D) Clark focal functional injury score and (E) glue remove time (*n* = 8). And ns means no significance between the ischemic 6 h group and the SA pretreatment + ischemic 6 h group. *means the SA pretreatment + ischemic 6 h group VS the EA pretreatment + ischemic 6 h group, *p < 0.05, **p < 0.01, ***p < 0.001, ****p < 0.0001. All data are presented as mean ± standard deviation.

**FIGURE 2:**
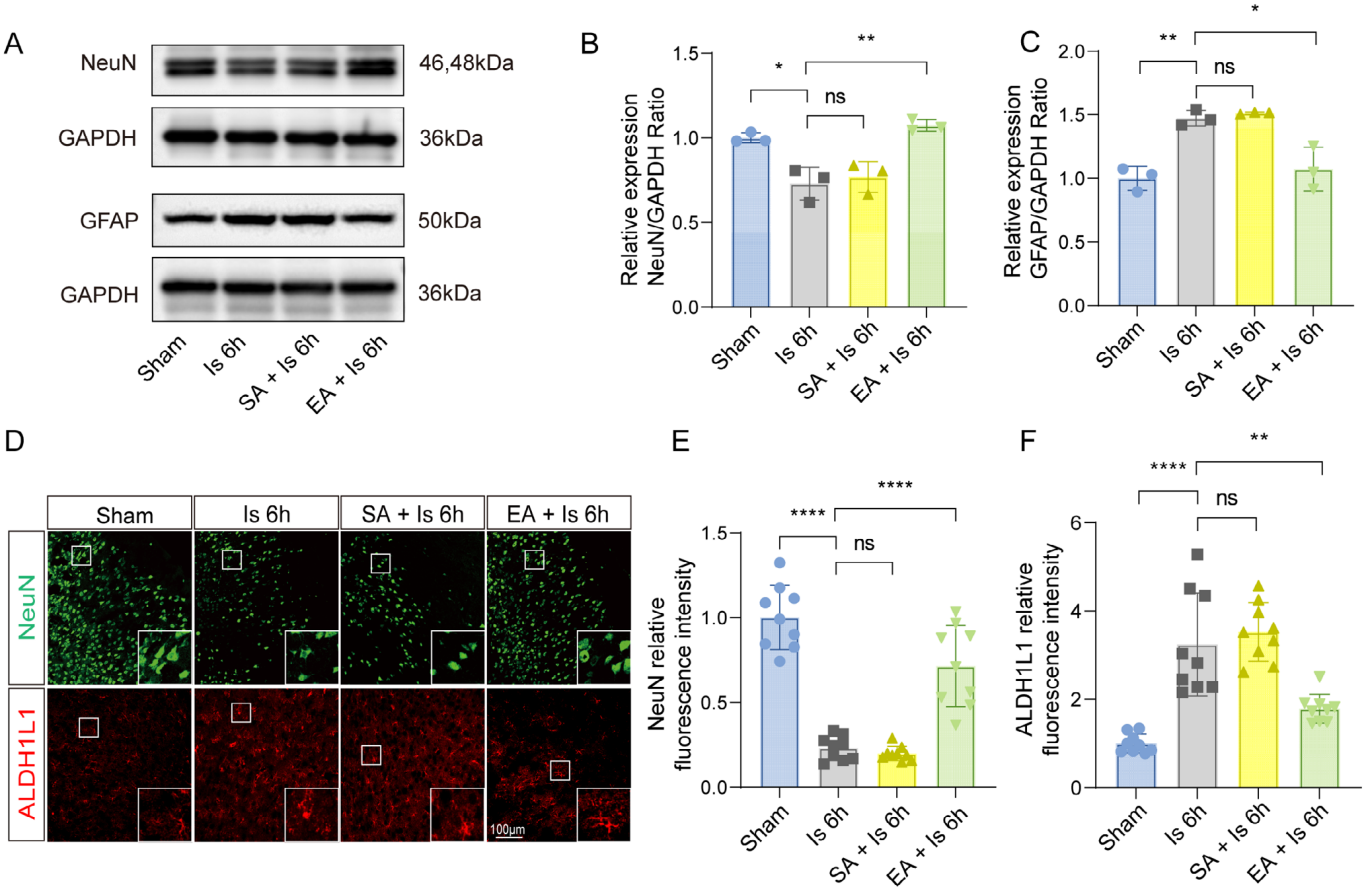
EA pretreatment mitigated brain injury in mice with ischemic stroke. (A–C) The ischemic brain levels of neuroglial markers of NeuN and astrocyte markers of GFAP were measured in the tissues using western blotting at 6 h post cerebral ischemia in mice with or without EA pretreatment (*n* = 3). (D) Representative image of IF staining with NeuN^+^ neuron (green) or ALDH1L1^+^ astrocytes (red) in cortex of ischemic mice compared to mice with EA pretreatment. (E–F) Statistic analysis of the relative fluorescence intensity of the NeuN and ALDH1L1 per field in cortex of mice with EA pretreatment compared to ischemic mice (*n* = 3; three fields per sample). Scale bar = 100 μm. ns, no significance; *p < 0.05, **p < 0.01, ***p < 0.001 and ****p < 0.0001. All data are presented as mean ± standard deviation.

First, to confirm the neuroprotective effects of EA pretreatment on the mice with ischemic stroke, we completed the experimental process according to the timeline shown in the diagram (Figure 1A). TTC staining was used to observe the volume of cerebral infarction areas and the results showed that there is a stable infarct area in mice at 6 h after pMCAO surgery, and a marked reduction of the infarcted volume was exhibited in mice with EA pretreatment than that in mice with sham EA (SA) pretreatment (Figure 1B,C). Meantime, Clark focal functional injury score (Figure 1D) and glue removal time (Figure 1E) increased to different degrees after modeling, and mice that were given EA pretreatment had lower neurological deficit scores compared to the SA pretreatment. Then, to observe the protective effect of EA pretreatment on changes at the cellular levels by detecting the neurons and astrocytes using western blot and IF staining. We found that the expression levels of NeuN, a neuronal marker, in the infarct area decreased compared to the sham group, which was significantly reversed in the mice that received EA pretreatment (Figure 2A,B,D,E). By contrast, the expression levels of glial fibrillary acidic protein (GFAP) or acetaldehyde dehydrogenase 1 L1 (ALDH1L1), both are markers of astrocytes, in the infarct region increased in response to cerebral ischemia, while such an increase at 6 h after pMCAO surgery was reduced by EA pretreatment (Figure 2A,C,D,F). These results indicate that EA pretreatment can reduce the brain injury of mice after cerebral infarction which is related to the increasing of neuronal survival and inhibition of astrocyte activation.

### 3.2 EA Pretreatment Downregulated Lactate Levels and Protein Kla Formation in Ischemic Brain Tissue of Mice After Ischemic Stroke

**FIGURE 3:**
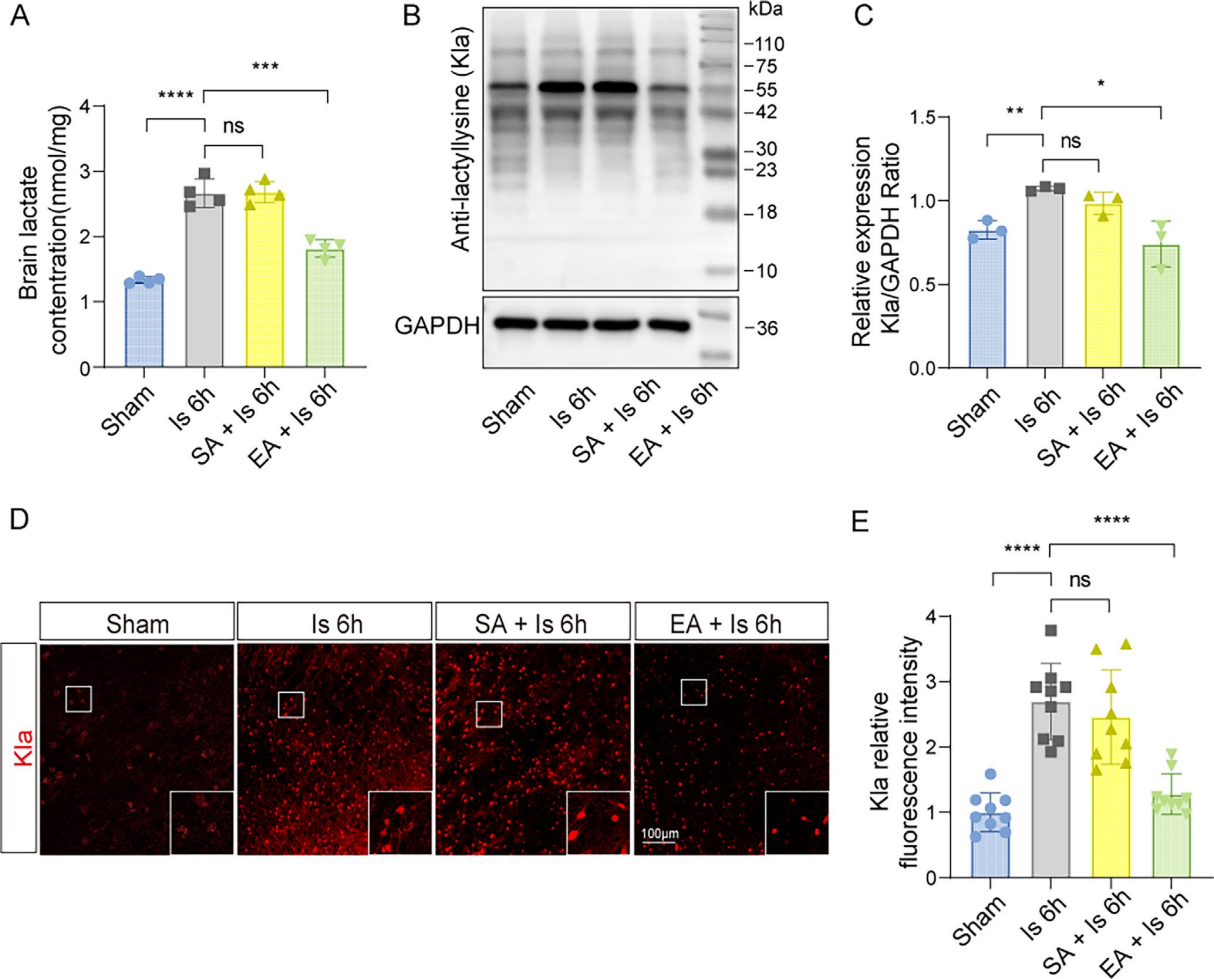
EA pretreatment downregulated lactate levels and Kla formation in ischemic brain tissue of mice after ischemic stroke. (A) Lactate levels in the ischemic brain at 6 h were measured in mice with or without EA pretreatment (*n* = 4). (B, C) The ischemic brain levels of markers of lysine lactylation modification of Kla were measured in the tissues using western blotting at 6 h post cerebral ischemia in mice with or without EA pretreatment (*n* = 3). (D) Representative image of IF staining with Kla in cortex of ischemic mice compared to mice with EA pretreatment. (E) Statistic analysis of the relative fluorescence intensity of the Kla per field in cortex of mice with EA pretreatment compared to ischemic mice (*n* = 3; three fields per sample). Scale bar = 100 μm. All data are presented as mean ± standard deviation. ns, no significance; *p < 0.05, **p < 0.01, ***p < 0.001 and ****p < 0.0001.

Next, we investigate the protective mechanisms of EA pretreatment on ischemic stroke. Given that we previously found that EA preconditioning could inhibit glycolysis in the ischemic brain tissues, which results in the downregulation of lactate production [23]. Moreover, our recent research showed that increased lactate contents drove the formation of protein Kla to aggravate the ischemic brain injury [26]. Based on this, then, we further confirmed whether EA pretreatment would reduce the production of lactate and the results showed that the increased levels of lactate in the ischemic brain tissues at 6 h were markedly reduced after EA pretreatment (Figure 3A). By performing the western blot and IF staining, we further found that there was also a significant reverse of the increased protein Kla levels at 6 h after cerebral ischemia in mice pretreated with EA (Figure 3B–E). These results suggest that EA pretreatment exerts neuroprotective effects on reducing ischemic brain injury by inhibiting the lactate‐derived formation of protein Kla.

### 3.3 EA Pretreatment Effectively Reversed the Aggravation of Ischemic Brain Injury Caused by Lactate Supplementation via Downregulating the Formation of Protein Kla

**FIGURE 4:**
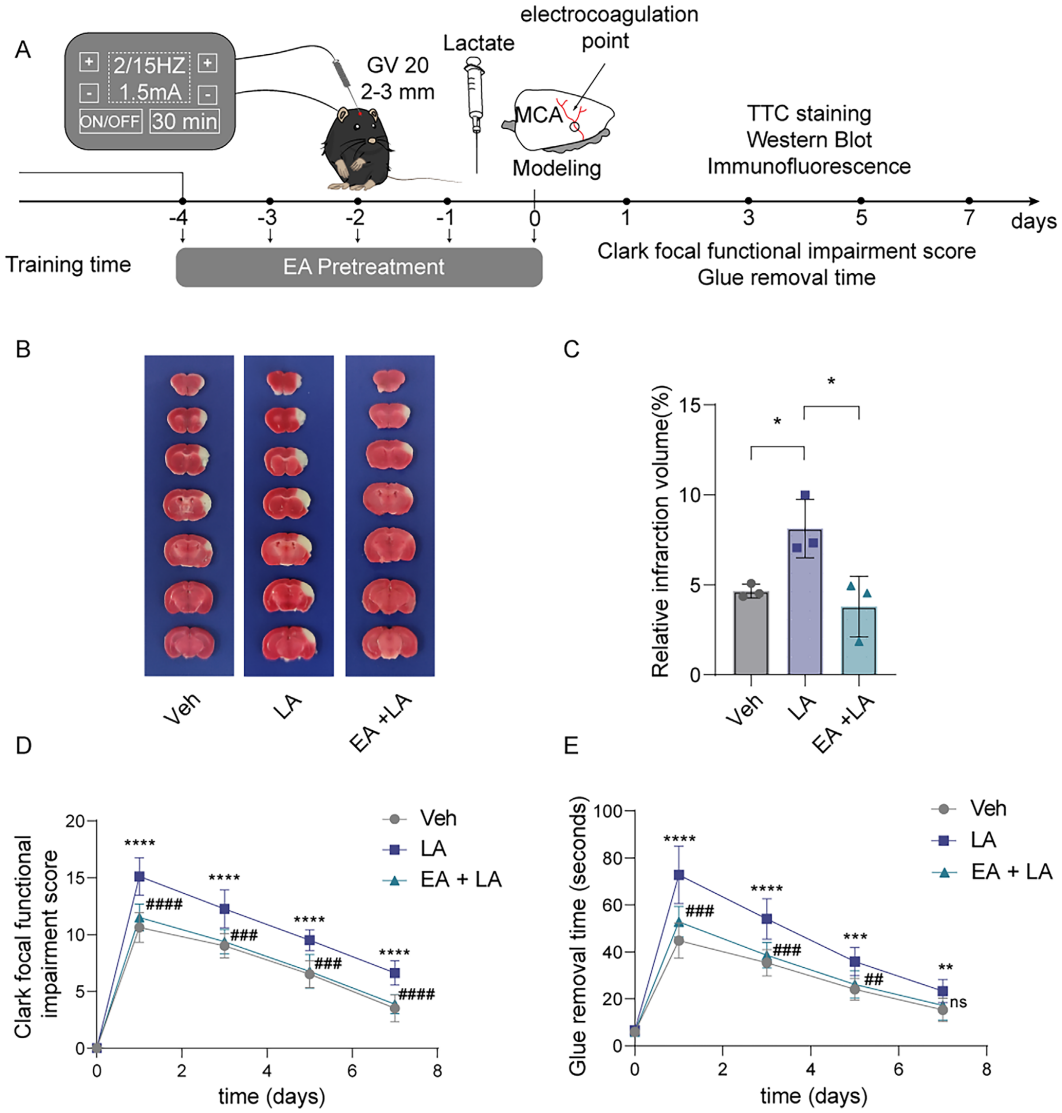
EA pretreatment effectively reversed the aggravation of ischemic brain injury caused by lactate supplementation via downregulating the formation of protein Kla. (A) The flowchart shows the modeling method, the timeline of EA pretreatment and lactate pretreatment, and the entire experimental design. (B) Representative images of 2,3,5‐triphenyltetrazolium chloride (TTC) staining of vehicle (saline), lactate and lactate + EA pretreatment after ischemia 24 h. The infarction area is white (*n* = 3). (C) Statistical chart of the proportion of cerebral infarction area. (D, E) Mice with lactate pretreatment and lactate + EA pretreatment show neurological function in tests of (D) Clark focal functional injury score and (E) glue remove time (*n* = 8). And *means the Veh group VS the LA group, *p < 0.05, **p < 0.01, ***p < 0.001, ****p < 0.0001. ^#^means the LA group VS the EA + LA group, ^#^ *p* < 0.05, ^##^ *p* < 0.01, ^###^ *p* < 0.001, ^####^ *p* < 0.0001. All data are presented as mean ± standard deviation. Veh indicates Vehicle.

To further clarify whether the neuroprotection of EA pretreatment on reducing ischemic brain injury is related to the downregulation of protein Kla, we then further enhanced the protein Kla levels by injecting exogenous lactate into the ischemic brain tissues of mice at 24 h before pMCAO surgery, meanwhile, the mice received EA pretreatment for five consecutive days (Figure 4A). After the extra lactate supplementation, the infarcted volumes were markedly increased at 6 h after cerebral ischemia, while such aggravation was reversed by the EA pretreatment (Figure 4B,C). Furthermore, the results of Clark focal functional injury score and glue removal time showed that EA pretreatment also reversed the aggravated neurological deficits within 7 days of mice with lactate supplementation before ischemic stroke (Figure 4D,E).

**FIGURE 5:**
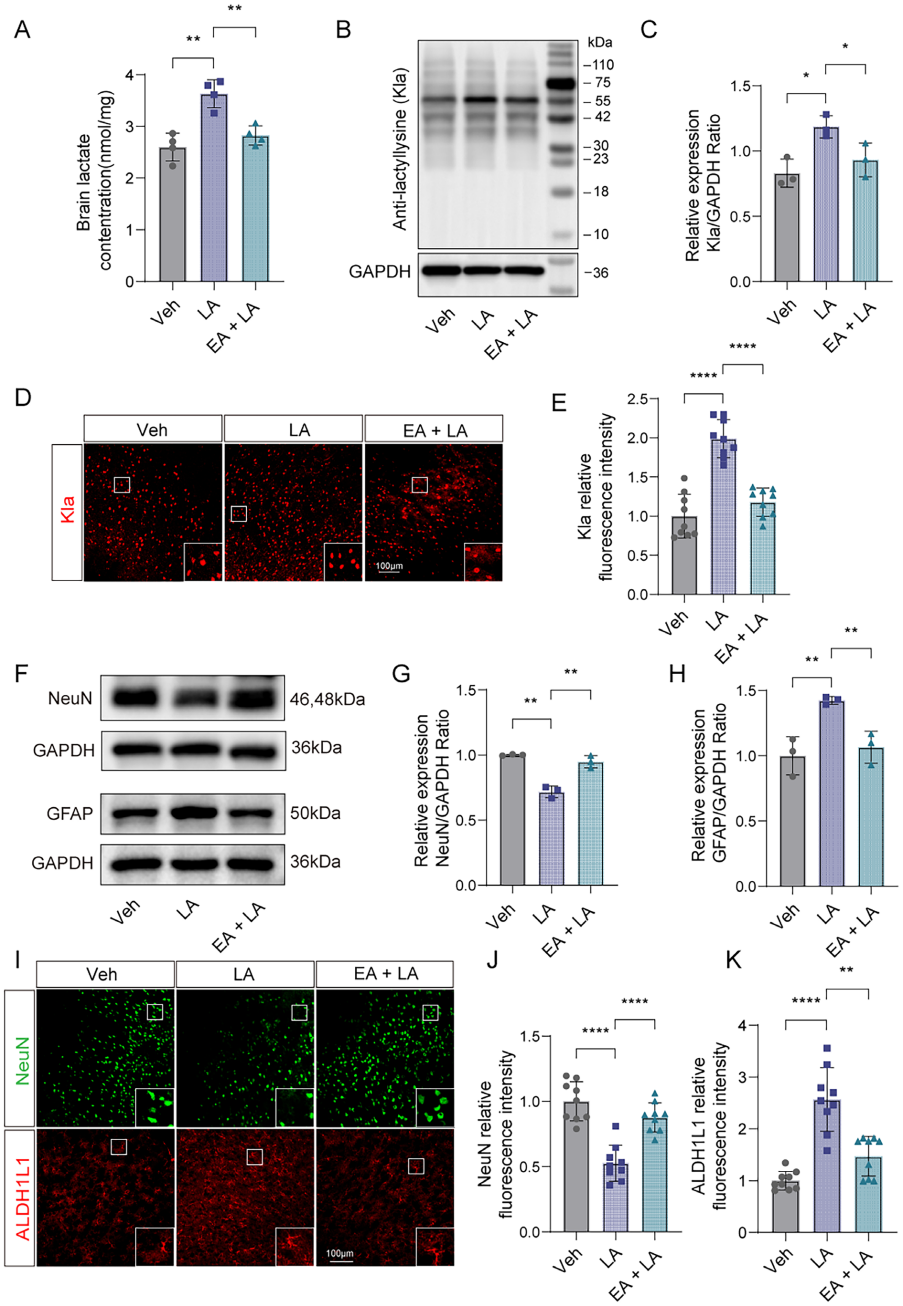
EA pretreatment effectively reversed the aggravation of ischemic brain injury caused by lactate supplementation via downregulating the formation of protein Kla. (A) Lactate levels in the brain with lactate pretreatment after ischemic 6 h were measured in mice with or without EA pretreatment (*n* = 4). (B, C) The ischemic brain levels of markers of lysine lactylation modification of Kla were measured in the lactate pretreatment brain tissues using western blotting at 6 h post cerebral ischemia in mice with or without EA pretreatment (*n* = 3). (D) Representative image of IF staining with Kla + in only lactate pretreatment cortex of ischemic mice compared to mice with lactate + EA pretreatment. (E) Statistic analysis of the relative fluorescence intensity of the Kla per field in cortex of mice with lactate + EA pretreatment compared to lactate pretreatment ischemic mice (*n* = 3; three fields per sample). (F–H) The ischemic brain levels of neuroglial markers of NeuN and astrocyte markers of GFAP were measured in the tissues using western blotting at 6 h post cerebral ischemia with lactate pretreatment in mice with or without EA pretreatment (*n* = 3). (I) Representative image of IF staining with NeuN^+^ neuron (green) or ALDH1L1^+^ astrocytes (red) in cortex of ischemic mice with lactate pretreatment compared to mice with lactate + EA pretreatment. (J, K) Statistic analysis of the relative fluorescence intensity of the Neun and ALDH1L per field in cortex of mice with lactate + EA pretreatment compared to lactate pretreatment only (*n* = 3; three fields per sample). Scale bar = 100 μm. ns, no significance; *p < 0.05, **p < 0.01, ***p < 0.001 and ****p < 0.0001 . All data are presented as mean ± standard deviation. Veh indicates Vehicle.

As expected, we found that the obviously increased lactate contents in ischemic mice with injection of exogenous lactate were also reduced by EA pretreatment (Figure 5A). Similarly, the lactate supplementation further increased protein Kla levels at 6 h in ischemic mice were also reduced after EA pretreatment evidenced by western blot and IF staining (Figure 5B–E). As before, we further examined the neuronal injury and death and astrocyte activation and found that exogenous lactate supplementation resulted in increased neuronal damage and activation of astrocytes at 6 h after cerebral ischemia, while EA pretreatment also reversed such increase in neuronal injury and astrocyte activation (Figure 5F–K). Taken together, these results indicate that EA pretreatment protects against ischemic brain injury via downregulating the levels of lactate and formation of protein Kla.

### 3.4 EA Pretreatment Failed to Further Reduce the Brain Injury of Mice With Ischemic Stroke After Glycolysis Inhibition by 2DG Supplementation

**FIGURE 6:**
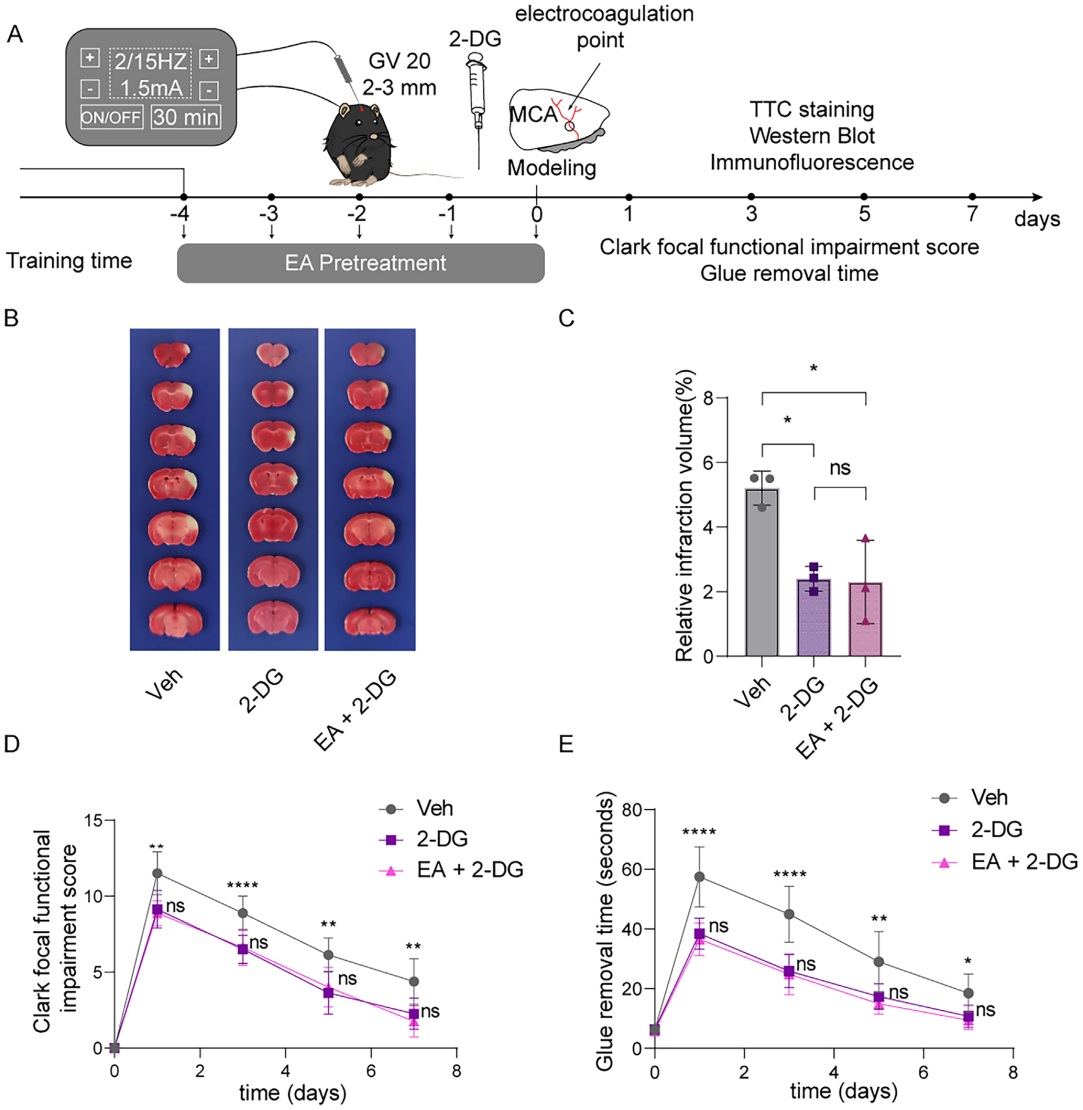
EA pretreatment failed to further reduce the brain injury of mice with ischemic stroke after glycolysis inhibition by 2DG supplementation. (A) The flowchart shows the modeling method, the timeline of EA pretreatment and 2‐DG pretreatment, and the entire experimental design. (B) Representative images of 2,3,5‐triphenyltetrazolium chloride (TTC) staining of vehicle (saline), 2‐DG and 2‐DG + EA pretreatment after ischemia 24 h. The infarction area is white (*n* = 3). (C) Statistical chart of the proportion of cerebral infarction area. (D, E) Mice with lactate pretreatment and 2‐DG + EA pretreatment show neurological function in tests of (D) Clark focal functional injury score and (E) glue remove time (*n* = 8). And *means the Veh group VS the 2‐DG group, *p < 0.05, **p < 0.01, ***p < 0.001, ****p < 0.0001. ^#^means the 2‐DG group VS the EA + 2‐DG group, ^#^ *p* < 0.05, ^##^ *p* < 0.01, ^###^ *p* < 0.001, ^####^ *p* < 0.0001. All data are presented as mean ± standard deviation. Veh indicates Vehicle.

**FIGURE 7:**
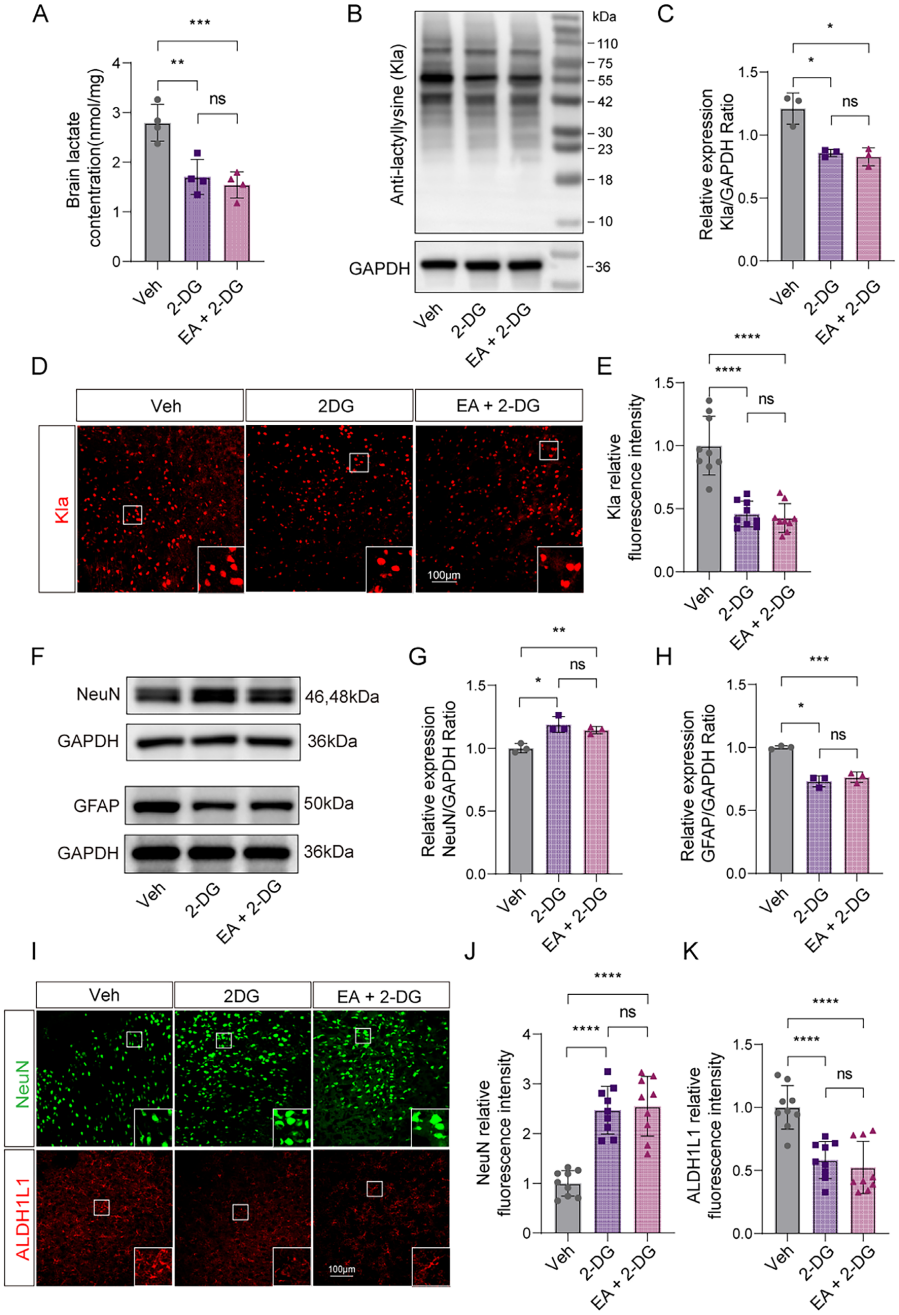
EA pretreatment failed to further reduce the brain injury of mice with ischemic stroke after glycolysis inhibition by 2DG supplementation. (A) Lactate levels in the brain with 2‐DG pretreatment after ischemic 6 h were measured in mice with or without EA pretreatment (*n* = 4). (B, C) The ischemic brain levels of markers of lysine lactylation modification of Kla were measured in the 2‐DG pretreatment brain tissues using western blotting at 6 h post cerebral ischemia in mice with or without EA pretreatment (*n* = 3). (D) Representative image of IF staining with Kla in only 2‐DG pretreatment cortex of ischemic mice compared to mice with 2‐DG + EA pretreatment. E Statistic analysis of the relative fluorescence intensity of the Kla per field in cortex of mice with 2‐DG + EA pretreatment compared to lactate pretreatment ischemic mice (*n* = 3; three fields per sample). (F–H) The ischemic brain levels of neuroglial markers of NeuN and astrocyte markers of GFAP were measured in the tissues using western blotting at 6 h post cerebral ischemia with 2‐DG pretreatment in mice with or without EA pretreatment (*n* = 3). I Representative image of IF staining with NeuN^+^ neuron (green) or ALDH1L1^+^ astrocytes (red) in cortex of ischemic mice with 2‐DG pretreatment compared to mice with 2‐DG + EA pretreatment. (J, K) Statistic analysis of the relative fluorescence intensity of the Neun and ALDH1L1 per field in cortex of mice with 2‐DG + EA pretreatment compared to 2‐DG pretreatment only (*n* = 3; three fields per sample). Scale bar = 100 μm. *p < 0.05, **p < 0.01, ***p < 0.001 and ****p < 0.0001. All data are presented as mean ± standard deviation. ns, no significance; Veh indicates Vehicle.

Next, we want to confirm whether EA pretreatment could further reduce the ischemic brain injury after inhibiting glycolysis. Then, after five consecutive EA pretreatment for mice, we administrated 2‐DG via intraperitoneal injection to inhibit glycolysis at 90 min before pMCAO surgery (Figure 6A). Unexpectedly, the results of TTC staining and neurological deficit scores both showed that EA pretreatment failed to further reduce the infarcted volume and neurological deficits of mice with ischemic stroke after glycolysis inhibition by 2DG supplementation (Figure 6B–E). Similarly, the EA pretreatment cannot further reduce lactate concentration (Figure 7A) and protein Kla levels (Figure 7B–E) based on inhibiting glycolysis by 2DG in the ischemic brain tissues of mice. Furthermore, the measurement of neuronal injury and death and astrocyte activation also showed the same results that EA pretreatment plus the inhibiting effects of 2DG no longer have extra effects on reducing neuronal injury and death and astrocyte activation at 6 h in ischemic brain tissues of mice (Figure 7F–K). These results strongly indicate that EA pretreatment protects against ischemic brain injury in mice via inhibiting lactate production and the formation of protein Kla.

## 4 Discussion

Ischemic stroke results in changes in energy metabolism in the local brain tissue due to ischemia and hypoxia. Under physiological conditions, mitochondria within neurons undergo aerobic respiration to provide energy [39]. While under pathological conditions of ischemia and hypoxia, neurons die rapidly because of the inhibition of mitochondrial respiration; in contrast, astrocytes utilize glycolytically generated ATP to increase their mitochondrial membrane potential, thus becoming more resistant to pro‐apoptotic stimuli [40]. Therefore, astrocytes were activated and proliferated after cerebral ischemia, and glycolysis was enhanced, which led to a large accumulation of lactate. Lactate plays a dual role in stroke brain injury by affecting different pathways [41, 42]. For example, a new study suggests that lactate transport between neurons and astrocytes is related to the sensitivity of neurons to ischemic stress [43]. However, it remains unclear how lactate plays a role in astrocytes or neuronal cells after cerebral ischemia brain injury, and how EA pretreatment exerts a neuroprotective effect based on the regulation of lactate content.

As we know, the damaged neuronal injury can be hardly repaired and the mammalian brain has been shown to possess a capacity of tolerance in response to cerebral ischemia [24], which hints us that minimizing neuronal damage is also a promising treatment. In addition, in this study, we want to investigate the metabolic mechanisms of cerebral ischemia regulated by EA. Considering the rapid changes in cerebral metabolism after ischemia, the posttreatment of EA may not in time regulate the metabolism of ischemic brain tissue. Therefore, we chose the pretreatment of EA which has been shown to regulate brain metabolism even in normal conditions, and which is neuroprotective for cerebral ischemia [23]. Thus, in this study, we performed EA pretreatment for ischemic mice. Furthermore, a number of studies have shown that EA pretreatment can alleviate brain injury caused by ischemia through anti‐inflammatory effect [44, 45], alleviated oxidative stress [46, 47], and regulating cellular autophagy [48, 49, 50]. These results strongly provide evidence that those people with a high risk of developing ischemic stroke could receive acupuncture treatment to enhance their brain tolerance.

The protein Kla is closely related to lactate content and has multiple functions in organisms. Such as histone kla modification which has powerful functions of regulating cell phenotype by regulating genes [51, 52], and inhibiting the ARF1 lactylation to alleviate neuronal damage caused by ischemia reperfusion [53], and lactate derived from astrocytes causing protein Kla in neurons, exacerbating brain ischemic injury [26]. The occurrence of Kla in different proteins may be involved in different stages of brain injury, and the specific protein Kla may play a neuroprotective role or neurotoxic role, which are unknown. So further application of mass spectrometry technology and bioinformatics analysis is needed to screen for highly expressed proteins Kla in neural cells and explore the specific functions and their relationship with EA pretreatment.

Therefore, our research focuses on whether brain injury caused by cerebral ischemia is associated with a process in which increased glycolysis is accompanied by astrocyte activation, producing large amounts of lactate, causing protein lactation, which leads to the death of nerve cells. There is hope to provide new therapeutic targets for the treatment of ischemic stroke and new ideas for improving the tolerance of neurons to ischemic conditions. However, taking into account the fact that it is not possible to intervene directly with the protein Kla, and can only be indirectly intervened through lactate: injecting 2DG or lactic acid. However, lactate also has other complex functions and many uncertain factors; so, the next step should be to choose more specific intervention methods to validate our conclusions. Finding out the mechanism by which high expression protein Kla after cerebral ischemia affects neurons or astrocytes is a question that we need to further clarify.

From our experimental results, EA pretreatment can reverse brain damage caused by increased lactate, but not further alleviate brain damage after injection of glycolysis inhibitors. Astrocytes are one of the main sites for glycogen breakdown and lactate release [54, 55]. Brain injury due to cerebral ischemia activates glycolysis in astrocytes, resulting in increased intracellular accumulation and release of lactic acid. This lactate accumulation promotes the synthesis of protein Kla, triggering complex pathological changes that may include enhanced autophagy and lysosomal degradation [56], affecting mitochondrial function and intracellular calcium balance [57], and ultimately leading to apoptosis of neuronal cells. The lactate content and protein Kla level decreased after EA pretreatment, indicating that EA pretreatment could inhibit the glycolysis process to a certain extent. When the glycolysis inhibitor 2‐DG is added, it already has an inhibitory effect on the glycolytic process, which may limit the further action space of EA pretreatment, so that the additive therapeutic effect cannot be achieved. This suggests that EA pretreatment does exert a protective effect by reducing the glycolytic pathway, thereby reducing lactate content and protein Kla levels.

In this study, we focus on whether EA pretreatment exerts neuroprotective effects by intervening in protein Kla. We first clarified the protective effect of EA pretreatment on brain injury in stroke and then found that EA pretreatment can exert neuroprotective effects on neurons and astrocytes by regulating lactate content and protein Kla. This study provides a new target for the treatment of brain injury caused by ischemic stroke and provides a new mechanism for EA pretreatment to exert neuroprotective effects.

In conclusion, we introduce the newly discovered lysine lactylation modification as a PTM method, starting from the energy metabolism disorders after cerebral infarction, to explore whether EA pretreatment can alleviate brain injury caused by cerebral infarction by regulating lactate content and protein Kla. At the same time, it indicates that the protein Kla can serve as a potential target to enhance the tolerance of brain neurons to ischemia and hypoxia.

## Author Contributions

X.‐Y.X., S.‐G.Y., and Y.T. contributed to the conception of the study. X.‐R.P. reviewed the literature and drafted the introduction. X.‐R.P., Y.‐D.Z., Y.‐H.G., J.‐H.Z., S.‐J.G., J.‐X.X., X.‐S.F., Y.‐F.W., X.‐X.Z., and P.‐F.W. performed the experiment and the data analysis. X.‐Y.X., S.‐G.Y., and Y.T. contributed to revise the manuscript. All authors contributed to the article and approved the submitted version.

## Ethics Statement

This study was reviewed and approved by the Institutional Animal Care and Use Committee of the Institute of Model Animals of Chengdu University of Traditional Chinese Medicine.

## Conflicts of Interest

The authors declare no conflicts of interest.

## Supporting information


Data S1.

## Data Availability

The raw data supporting the conclusions of this article will be made available by the authors, without undue reservation.
