# Supplementary material for: Electroacupuncture Pretreatment Reduces Ischemic Brain Injury by Inhibiting the Lactate Production and Its Derived Protein Lactylation Formation
Source: CNS Neurosci Ther. 2025 Jan 20;31(1):e70231. doi: 10.1111/cns.70231 (PMC11746925; doi:10.1111/cns.70231)

Full unedited blot for Figure 2A

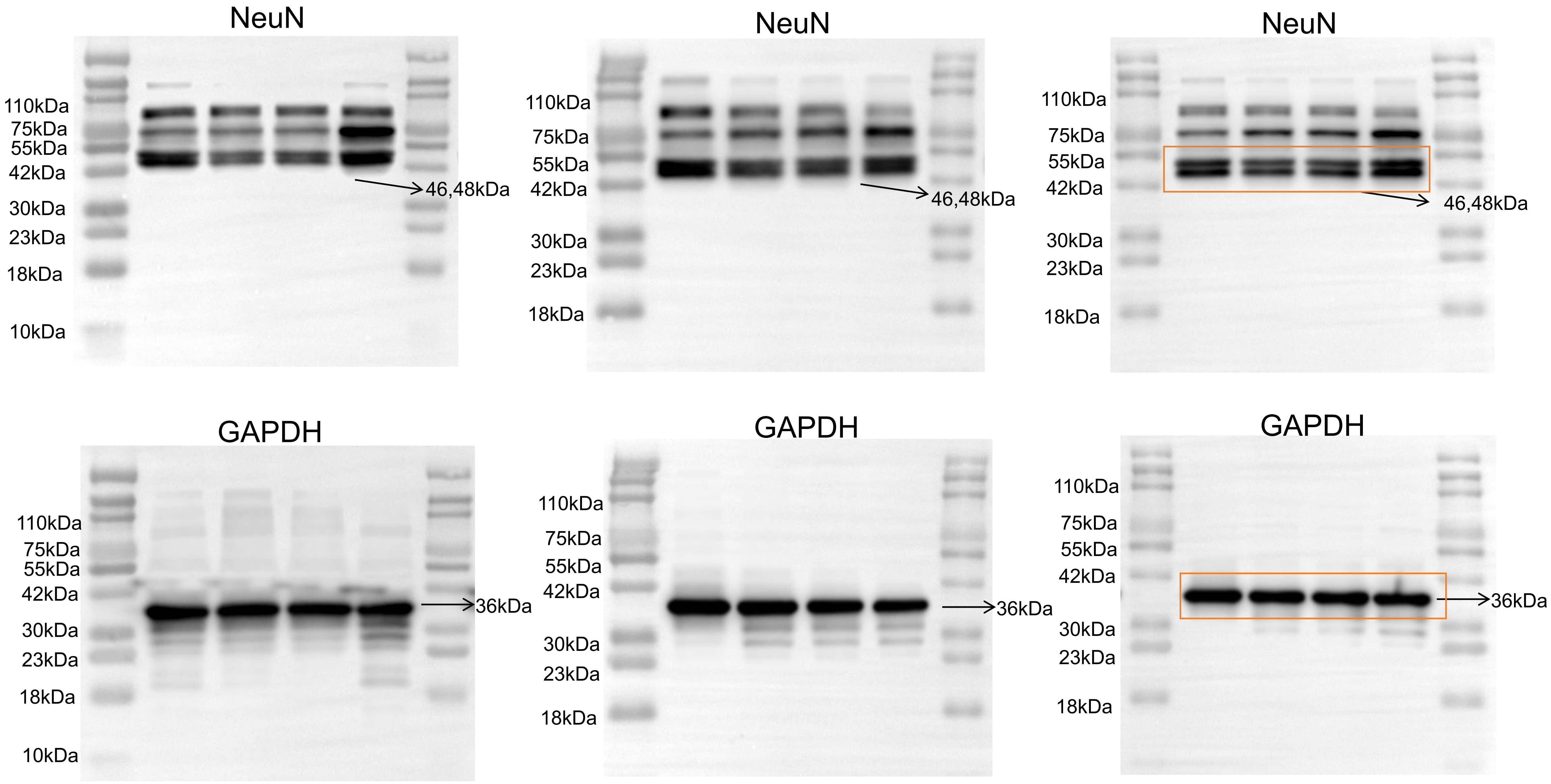

Full unedited blot for Figure 2A

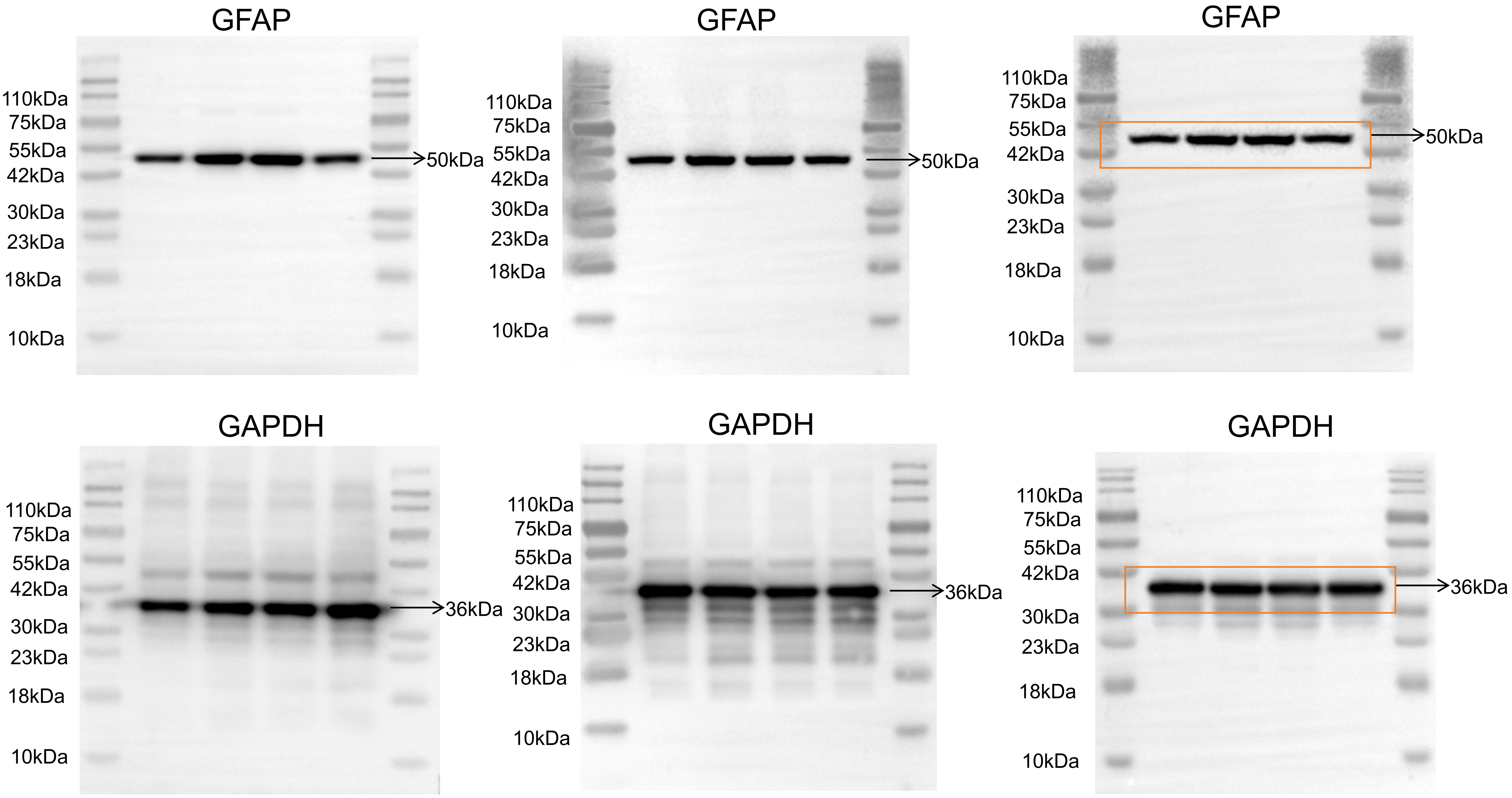

Full unedited blot for Figure 3B

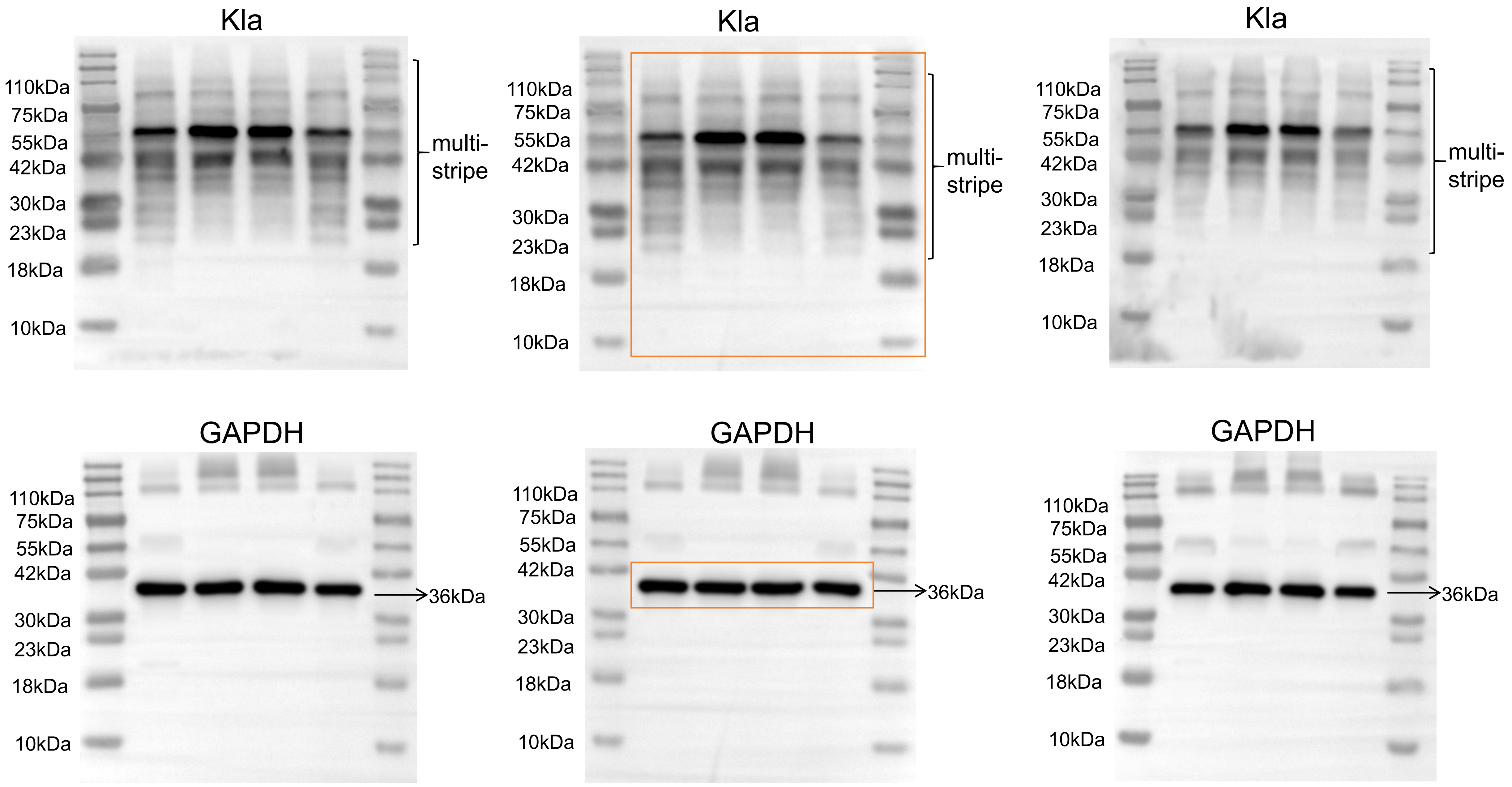

Full unedited blot for Figure 5B

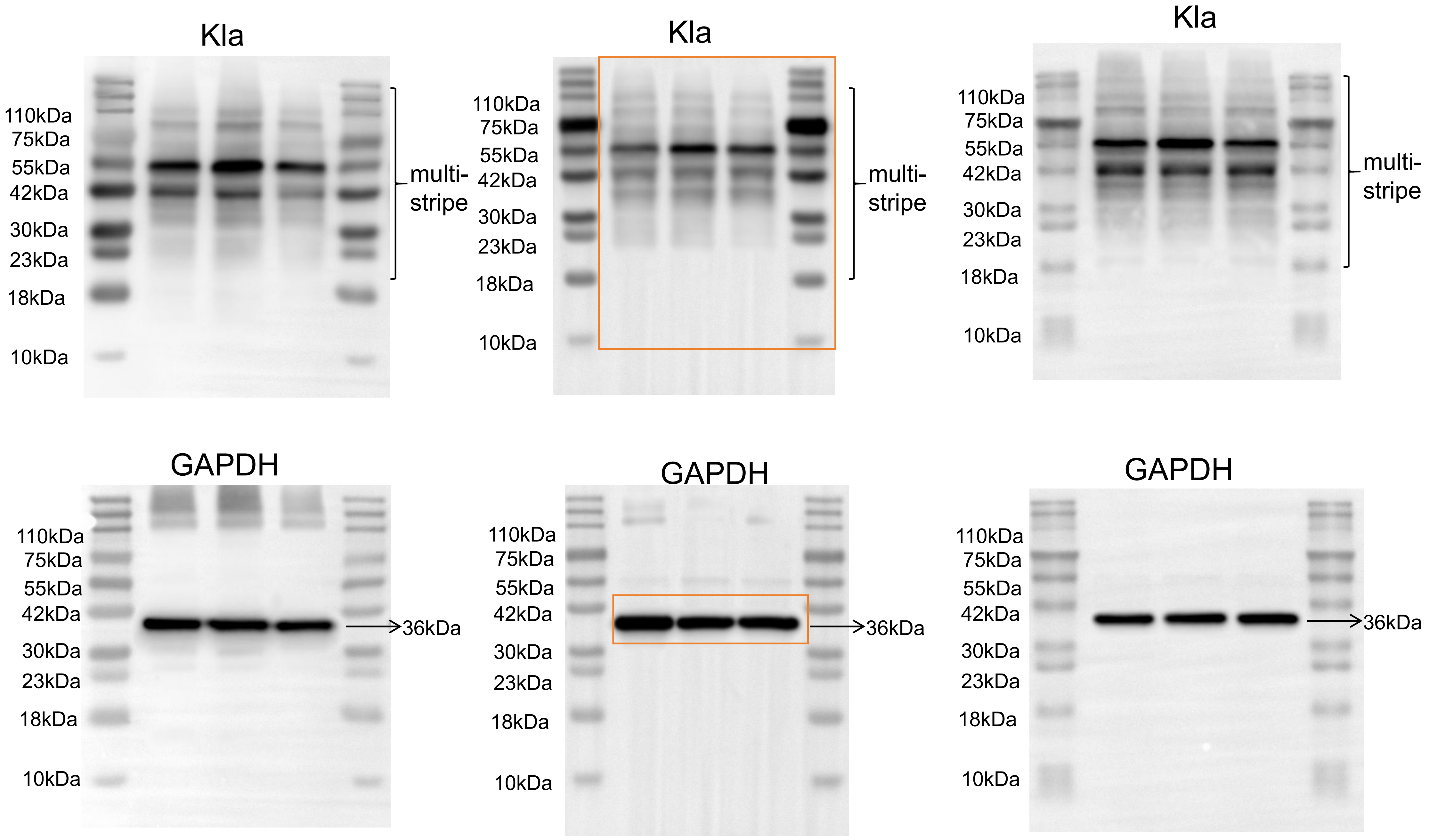

Full unedited blot for Figure 5F

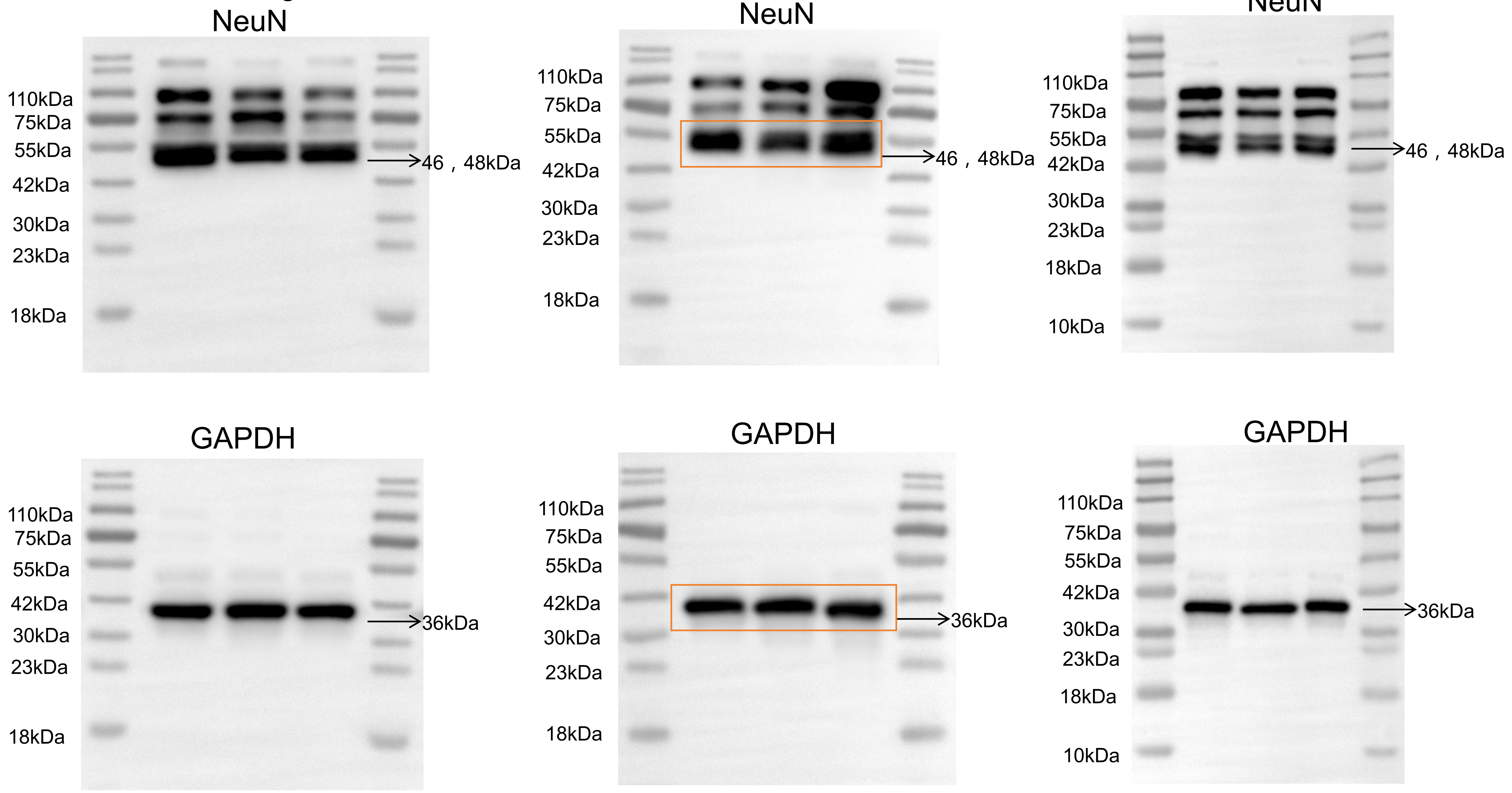

Full unedited blot for Figure 5F

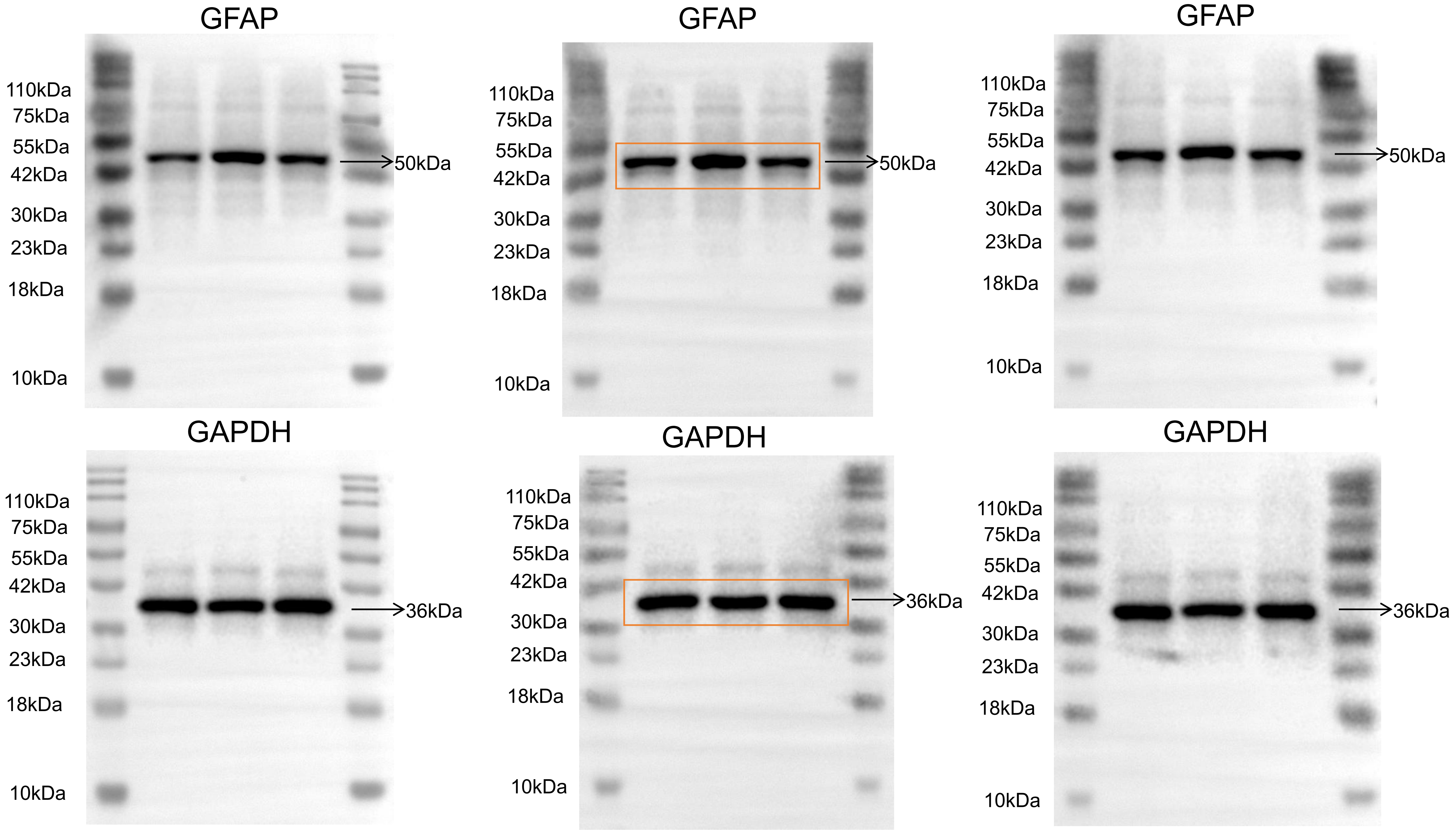

Full unedited blot for Figure 7B

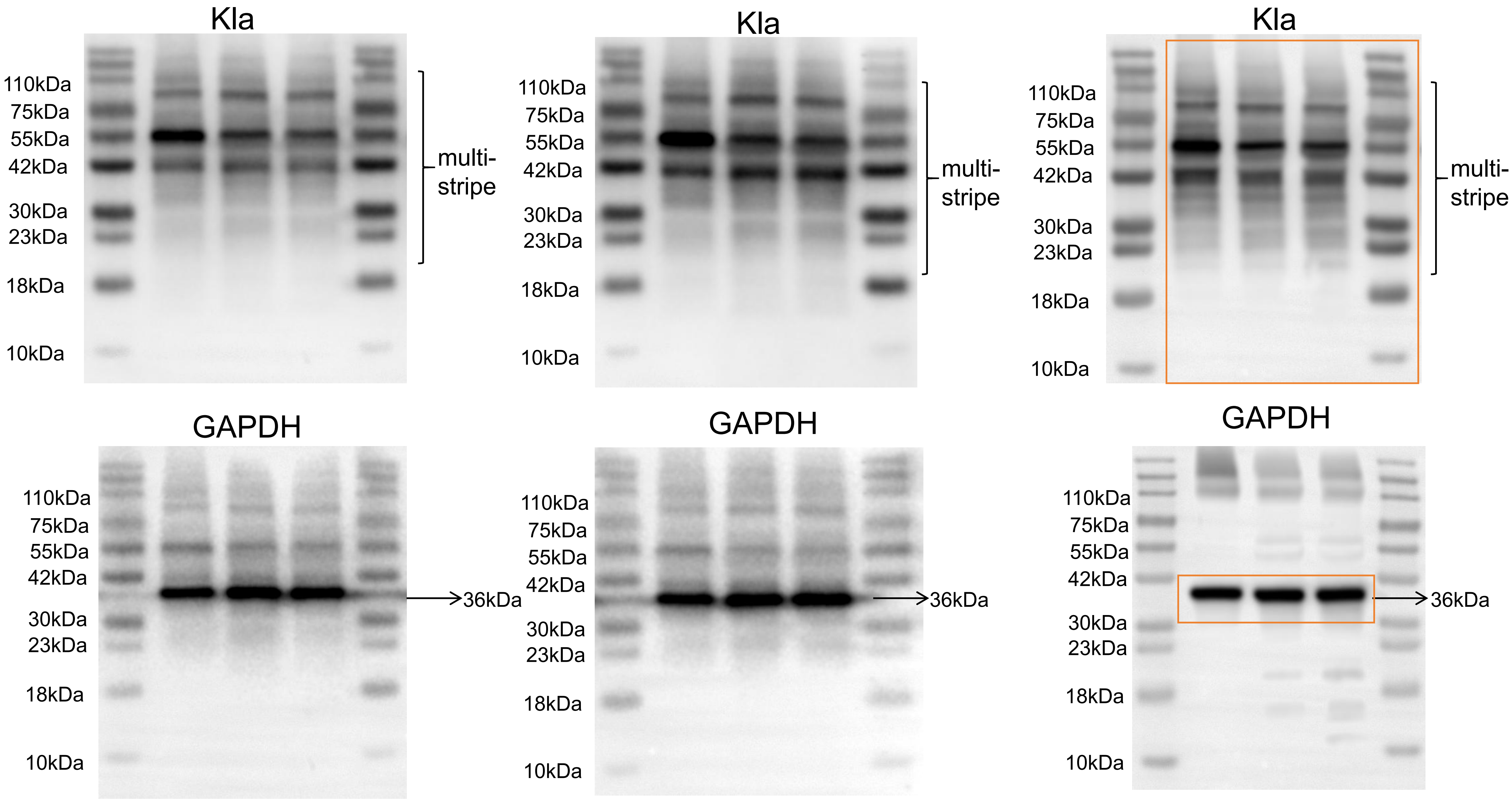

Full unedited blot for Figure 7F

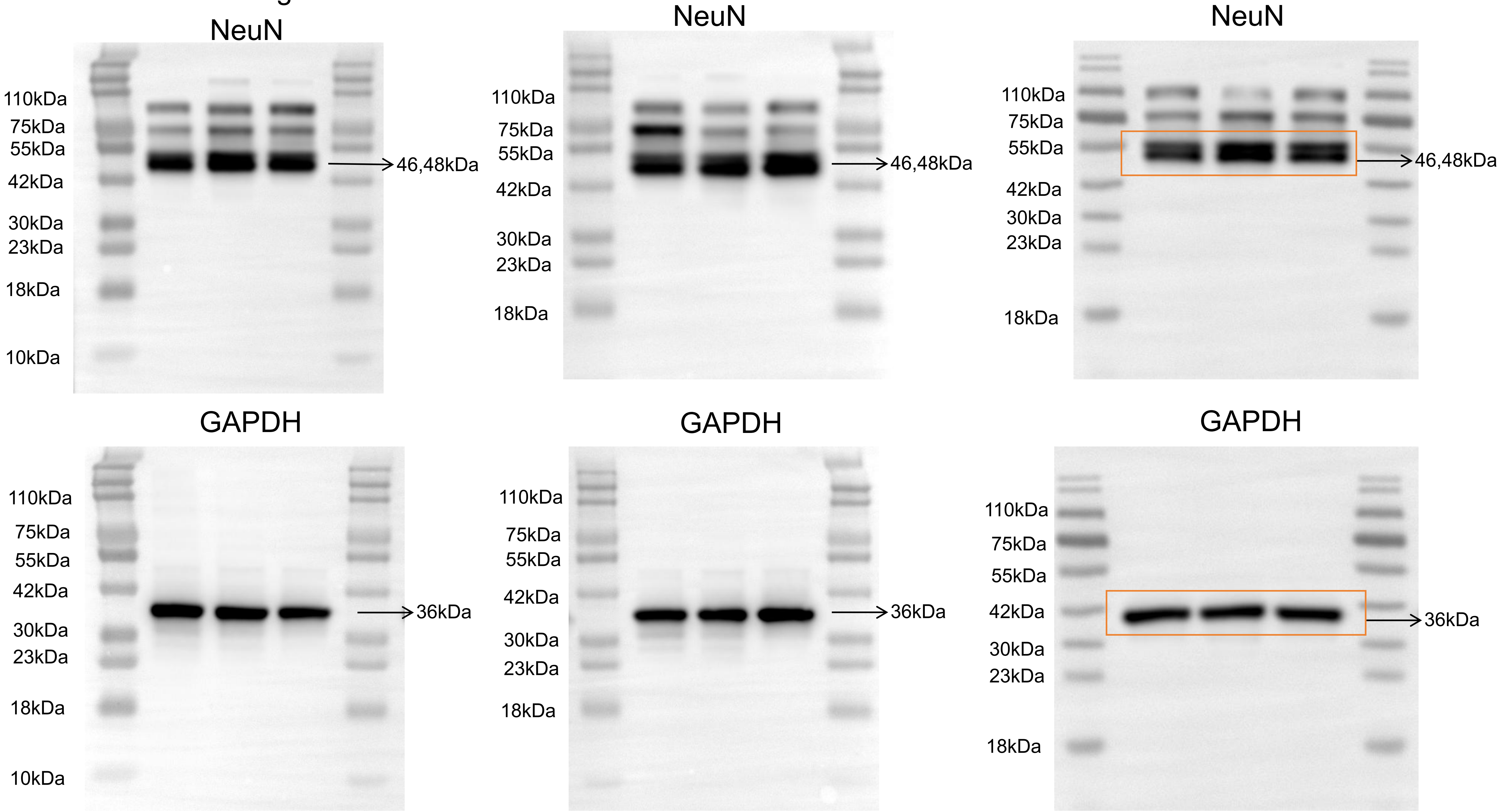

Full unedited blot for Figure 7F

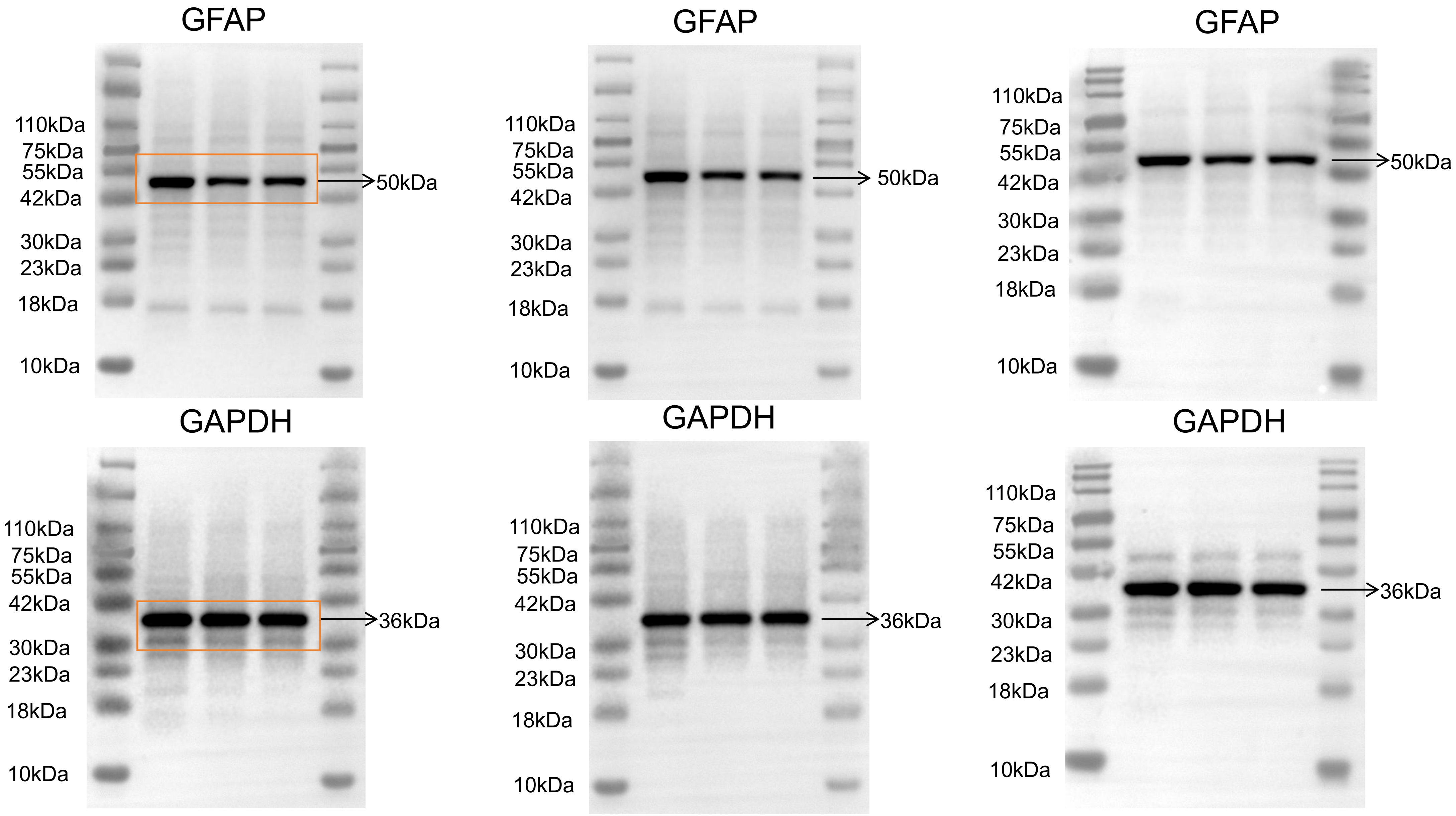

Supplement: Supplementary file 1 — Data S1. [file CNS-31-e70231-s001.pdf]
